# Generating Operative Workflows for Vestibular Schwannoma Resection: A Two-Stage Delphi's Consensus in Collaboration with the British Skull Base Society. Part 1: The Retrosigmoid Approach

**DOI:** 10.1055/a-1886-5500

**Published:** 2022-10-10

**Authors:** Hugo Layard Horsfall, Danyal Z. Khan, Justin Collins, Stephen Cooke, Simon R. Freeman, Nihal Gurusinghe, Susie Hampton, Carl Hardwidge, Richard Irving, Neil Kitchen, Andrew King, Sherif Khalil, Chan H. Koh, Colin Leonard, Hani J. Marcus, William Muirhead, Rupert Obholzer, Omar Pathmanaban, Iain J. A. Robertson, Jonathan Shapey, Danail Stoyanov, Mario Teo, James R. Tysome, Shakeel R. Saeed, Patrick Grover

**Affiliations:** 1 Victor Horsley Department of Neurosurgery, National Hospital for Neurology and Neurosurgery, London, United Kingdom; 2 Wellcome/EPSRC Centre for Interventional and Surgical Sciences, University College London, London, United Kingdom; 3 Department of Urooncology, University College London Hospitals National Health Service Foundation Trust, London, United Kingdom; 4 Department of Neurosurgery, Belfast Health and Social Care Trust, Belfast, United Kingdom; 5 Department of Otolaryngology, Manchester Centre for Clinical Neurosciences, Salford Royal Hospital, Salford, United Kingdom; 6 Department of Neurosurgery, Lancashire Teaching Hospital, Preston, United Kingdom; 7 Department of Ear, Nose and Throat, Belfast Health and Social Care Trust, Belfast, United Kingdom; 8 Department of Neurosurgery, University Hospital Sussex, Brighton, United Kingdom; 9 Department of Ear, Nose and Throat, Queen Elizabeth Hospital, Birmingham, United Kingdom; 10 Geoffrey Jefferson Brain Research Centre, Manchester Academic Health Science Centre, Manchester, United Kingdom; 11 Northern Care Alliance National Health Service Group, University of Manchester, Manchester, United Kingdom; 12 The Royal National Throat, Nose and Ear Hospital, London, United Kingdom; 13 Department of Neurosurgery, Manchester Centre for Clinical Neurosciences, Salford Royal Hospital, Salford, United Kingdom; 14 Department of Neurosurgery, Nottingham University Hospitals, Nottingham, United Kingdom; 15 Department of Neurosurgery, Kings College Hospital, London, United Kingdom; 16 Bristol Institute of Clinical Neuroscience, Southmead Hospital, Bristol, United Kingdom; 17 Department of Ear, Nose and Throat, Cambridge University Hospitals, Cambridge, United Kingdom

**Keywords:** retrosigmoid, translabyrinthine, vestibular schwannoma, skull base surgery, consensus, Delphi

## Abstract

**Objective** An operative workflow systematically compartmentalizes operations into hierarchal components of phases, steps, instrument, technique errors, and event errors. Operative workflow provides a foundation for education, training, and understanding of surgical variation. In this Part 1, we present a codified operative workflow for the retrosigmoid approach to vestibular schwannoma resection.

**Methods** A mixed-method consensus process of literature review, small-group Delphi's consensus, followed by a national Delphi's consensus, was performed in collaboration with British Skull Base Society (BSBS). Each Delphi's round was repeated until data saturation and over 90% consensus was reached.

**Results** Eighteen consultant skull base surgeons (10 neurosurgeons and 8 ENT [ear, nose, and throat]) with median 17.9 years of experience (interquartile range: 17.5 years) of independent practice participated. There was a 100% response rate across both Delphi's rounds. The operative workflow for the retrosigmoid approach contained three phases and 40 unique steps as follows: phase 1, approach and exposure; phase 2, tumor debulking and excision; phase 3, closure. For the retrosigmoid approach, technique, and event error for each operative step was also described.

**Conclusion** We present Part 1 of a national, multicenter, consensus-derived, codified operative workflow for the retrosigmoid approach to vestibular schwannomas that encompasses phases, steps, instruments, technique errors, and event errors. The codified retrosigmoid approach presented in this manuscript can serve as foundational research for future work, such as operative workflow analysis or neurosurgical simulation and education.

## Introduction

Vestibular schwannomas are typically resected through one of the following three approaches: (1) retrosigmoid, (2) translabyrinthine, and (3) middle fossa. 1 The retrosigmoid and translabyrinthine are the most commonly utilized approaches and provide good outcomes relating to safety and efficacy profiles. 1 2 3 The middle fossa approach is rarely performed in the United Kingdom due to the high risks of damage to the facial nerve and seizures caused by temporal lobe manipulation. 4 5 6 There is variability between surgeons and centers on how to perform the operation, based on surgeon preference and training, tumor location, and characteristics, all of which may result in differing surgical outcomes. 1 6 7 Additionally, lateral skull base procedures are technically challenging, have steep learning curves, and centers have varying degrees of collaboration with ear, nose, and throat (ENT) surgeons for different parts of the operation.

An initial step to understanding how an operation is performed is to deconstruct an operation and create a common language. A technique to systematically deconstruct complex procedures into defined tasks and errors is known as “operative workflow analysis.” 8 9 The surgical procedure is broken down into phases which contain a series of steps, generating a workflow framework. 9 During each step (e.g., suturing), surgical instruments (e.g., forceps) are used to perform maneuvers (e.g., knot tying) via a series of gestures (e.g., grasping and pulling suture). 10 Similarly, at each step, there is the potential for technical errors, lapses in surgical technique, and adverse events, an event which may lead to adverse outcomes or postoperative complications. 9 Deconstructing a complex procedure into a systematic operative workflow requires expert consensus. Existing literature has demonstrated subject experts generating comprehensive and standardized workflow framework for nonneurosurgical proceedures 11 12 13 and more recently a neurosurgical procedure. 14 The Delphi technique allows the generation of group consensus through iterative questionnaires/surveys, interspersed with feedback. 13

The management of vestibular schwannomas has benefitted from international, multidisciplinary consensus statements relating to stereotactic radiosurgery, 15 reporting outcomes, 16 and, more recently, large vestibular schwannomas. 17 Currently, there is no consensus on the operative workflow for the retrosigmoid or translabyrinthine approaches for vestibular schwannomas. Expert, consensus-driven operative workflows can provide multiple benefits: (1) workflow analysis; (2) training; (3) creation of high-fidelity simulation models; (4) objective assessment of procedure-specific surgical skills; (5) evaluation of novel technologies or techniques; (6) operating room efficiency improvements. 9 11 18 19

We created an operative workflow for the retrosigmoid approach for vestibular schwannoma, through an expert consensus process in collaboration with the British Skull Base Society (BSBS). This operative workflow aimed to digitize the approaches and provide foundational research in which to build, for example, the application of artificial intelligence to vestibular schwannoma resection.

## Methods

### Overview

**Fig. 1:**
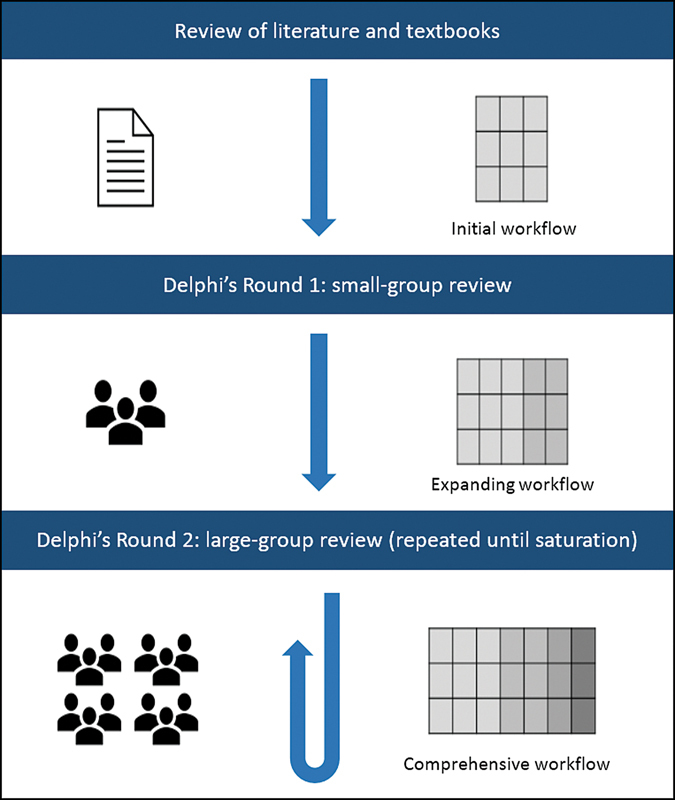
Schematic diagram of Delphi's process, highlighting the generation of a surgical workflow through iterative consensus from British Skull Base Society expert members. 20 Adapted from Marcus et al. 14

The methodology was drawn from previous work from our group. 14 20 This process aimed to generate a comprehensive workflow framework which captured how each approach could reasonably be performed. We did not aim to dictate how an operation should be done. The beginning of the operation was taken as the first incision, adhering to the American College of Surgeon's definition of surgery, “structurally altering the human body by the incision or destruction of tissues.” 21 22 Therefore, variation relating to position of the patient and incision analysis was not within the scope of this work, although the authors recognize that positioning plays a critical role for any given procedure. The components for workflow analysis and associated definitions are listed in Table 1 . Expert input will be derived through an iterative, mixed-methods consensus process ( Fig. 1 ).

**Table 1:** Definition of operative workflow terminology per domain

Domain	Definition	Example
Phase	A major event occurring during a surgical procedure, composed of several steps 6	Approach and exposure - encompassing the beginning of surgery until tumor debulking
Step	A sequence of activities used to achieve a surgical objective 24	Seal mastoid air cells
Instrument	A tool or device for performing specific actions (such as cutting, dissecting, grasping, holding, retracting, or suturing) during a surgical step	Bone wax
Technical error	Lapses in operative technique while performing a surgical step 25	Failure to seal mastoid air cells
Adverse event	An intraoperative event which is a result of a technical error and has the potential to lead to a post-operative adverse outcome/complication 25	Cerebrospinal fluid rhinorrhea

### Modified Delphi's Process and Sampling

#### Literature Review

We performed a literature review of Greenberg's Handbook of Neurosurgery, Youmans and Winn Neurological Surgery, and Operative Cranial Neurosurgical Anatomy, and undertook a PubMed and EMBASE search using the keywords “retrosigmoid,” “translabyrinthine,” and “vestibular schwannoma resection” 1 2 3 ( Fig. 1 ).

#### Delphi's Round 1

The initial literature-based operative workflow was reviewed by a group of five consultant skull base surgeons, including neurosurgery and ENT, based at the National Hospital for Neurology and Neurosurgery, London, United Kingdom. Each consultant surgeon reviewed the operative workflow individually via computerized document with the definitions of phases, steps, instruments, technical errors, and adverse events as above ( Table 1 ). Each expert was asked a series of questions via e-mail, seeking to assess the completeness and accuracy of the workflow ( Supplementary Material A , available in the online version). 14 Any additional suggestions were reviewed and added to the workflow matrix if in scope and not duplicate. According to the Delphi technique, circulation and iterative revision of the workflow was repeated until data saturation was achieved, that is, all experts were satisfied that the operative workflow was complete and accurate. 14

#### Delphi's Round 2

The refined workflow was circulated nationally with skull base surgeons (neurosurgeons and ENT) who were members of the BSBS, 25 the United Kingdom and Ireland's society primarily focused on skull base pathology. The entirety of the BSBS was invited to participate via e-mail. All contributing authors are specialist of lateral skull base surgeons with an independent surgical practice in vestibular schwannoma surgery who are members of the BSBS (either neurosurgery or ENT). Consultant surgeon members from the BSBS were asked to assess the workflow and suggest any amendments to encompass possible variation in practice and technique. Additional suggestions were reviewed and added to the workflow if (1) in scope and (2) not duplicate. 14 Round 2 was completed until all surgeons agreed that the workflow captured the operative practice and that there were no additional suggestions for the workflow from the participant group. Both the retrosigmoid and translabyrinthine approaches were completed in parallel: surgeons within the BSBS were given the opportunity to contribute to either approach depending on their personal clinical practice and expertise. Experience for all authors was calculated from the date they were added to the General Medical Council's Specialist Register, a list of doctors who have completed their postgraduate training and eligible to work as a consultant. 26

### Administration

Invitations to participate in the Delphi's process were sent via direct e-mail only. Workflow documents were presented using Microsoft Word (Version 16.4, Microsoft, United States) in both rounds and supported by Google Forms in Round 2 (Google LLC, United States).

### Data Collection and Analysis

Participant demographics collected included surgical specialty and unit. The collected data regarding the surgical workflow were quantitative (whether participants agree that it is complete and accurate) and qualitative (additional suggestions or comments). 14 Content analysis was used to analyze free-text responses: to remove out-of-scope suggestions, group similar suggestions together, and compare them to existing data points in the workflow. Data analysis and workflow updates were performed in duplicate by two independent analyzers (H.L.H. and P.G.).

### Ethics

This study is independent of national health services and does not require ethical approval – interrogated via online Health Research Authority decision tool ( Supplementary Material B , available in the online version). 27 26

## Results

### Participants

The Delphi Round 1 was completed by a group of five consultant skull base surgeons. Two neurosurgeons at the National Hospital for Neurology and Neurosurgery, London, United Kingdom, and three ENT surgeons at the Royal National Throat, Nose, and Ear Hospital, London, United Kingdom. Cumulatively, they had a median of 12.3 years and interquartile range (IQR) 16.0 years of experience (IQR: 1 9.6 years; IQR: 3 25.5 years). The Delphi Round 1 was repeated four times during a 4-month period (October 2020–February 2021) until saturation.

The Delphi Round 2 was completed by 10 neurosurgeons and 8 ENT surgeons based at 11 centers across the United Kingdom. All contributing authors are specialist lateral skull base surgeons with an independent surgical practice in vestibular schwannoma surgery who are members of the BSBS (either neurosurgery or ENT). Cumulatively, they had a median of 17.9 years and IQR of 17.5 years of experience (IQR: 1 8.0 years; IQR: 3 25.5 years). Round 2 was repeated twice during a 3-month period (May–July 2021) until saturation. There was a 100% response rate and no attrition across both the Delphi Rounds.

### Retrosigmoid Approach

Three distinct operative phases were delineated: (1) approach and exposure, (2) tumor debulking and excision, and (3) closure. The operative workflow had 40 unique steps. Preoperative set-up and postoperative protocols were recognized as important but not included as per the defined study scope.

#### Phase 1: Approach and Exposure

This phase consisted of 10 steps from retroauricular incision, approaching the cerebellopontine angle, and dissection of the arachnoid plane from the tumor capsule ( Table 2 ).

**Table 2:** Retrosigmoid operative workflow phase 1: approach and exposure

No.	Steps	Instruments	Technique error	Event error
1	Retroauricular incision to bone ± pericranial graft	Scalpel, monopolar, retractors	•Vertebral artery injury •Soft tissue dissection too far lateral	•Vertebral artery bleeding or infarct •Laceration
2	Hemostasis	Monopolar, bipolar, suction, bone wax		
3	± Retrosigmoid craniectomy ± collection of bone dust	Cutting burr, Kerrison's punch, periosteal elevator, bone wax	•Dural sinus injury •Opening of mastoid air cells without repair	•Hemorrhage •Air embolism •Sinus thrombosis •CSF rhinorrhea
4	± Retrosigmoid craniotomy	Perforator, Penfield's dissector, McDonald's dissector, matchstick burr, cutting burr, craniotome, bone wax	•Dural sinus injury •Opening of mastoid air cells without repair	•Hemorrhage •Air embolism •Sinus thrombosis •CSF rhinorrhea
5	Seal mastoid air cells	Bone wax	•Failure to seal mastoid air cells	•CSF rhinorrhea
6	Durotomy	Scalpel, blunt hook, Cottonoid patties, dural scissors	•Dural sinus injury	•Hemorrhage •Air embolism •Sinus thrombosis
7	Suture to dural edges	Suture	•Dural sinus injury	•Hemorrhage •Air embolism •Sinus thrombosis
8	Cisterna magna opening	Microscope, brain retractor, scalpel, sharp hook, Cottonoid patties	•Failure to open cisterna magna •Insufficient CSF egress •Excessive retraction	•Cerebellar swelling and retraction injury •Hemorrhage
9	Approach to cerebellopontine angle and retraction of cerebellum	Microscope, microdissector, microscissors, suction, retractors, linteens, Cottonoid patties, rubber dam	•Stretching of cranial nerves •Excessive retraction	•CN VII, XI, X palsy •Superior petrosal vein injury •Tearing of bridging veins and hemorrhage •Cerebellar swelling and retraction injury
10	Dissection of arachnoid plane from tumor capsule	Microscope, bipolar, suction, microdissector, microscissors, Cottonoid patties, nontoothed bayonet fine tip forceps	•Loss of arachnoid plane or entry into incorrect plane •Excessive traction on capsule	•Hemorrhage •CN injury

Abbreviations: CFS, cerebrospinal fluid; CN, cranial nerve.

#### Phase 2: Tumor Debulking and Excision

This phase consisted of 21 steps, starting with identification of the facial nerve using a stimulator, tumor debulking at the superior, and inferior poles, with lateral–medial and medial–lateral dissection, and culminating with stepwise rolling and debulking of the tumor ( Table 3 ). It is acknowledged that the exact order of the tumor debulking is surgeon and tumor characteristic dependent. As such, each operation will contain the steps listed within this phase, but perhaps in a different order as written depending on intraoperative findings. Further, facial nerve reanimation may or may not take place intraoperatively, and the type of nerve graft used is surgeon dependent.

**Table 3:** Retrosigmoid operative workflow phase 2: tumor debulking and excision

No.	Steps	Instruments	Technique error	Event error
1	Posterior aspect of tumor stimulated for facial nerve	Microscope, facial nerve stimulator	•Failure to identify CN VII	•CN VII palsy
2	Tumor capsule opened and primary debulking	Microscope, facial nerve stimulator, bipolar, suction, microdissector, Cottonoid patties, ultrasonic aspirator, tumor holding forceps, rongeur	•Incomplete hemostasis •CN injury	•Hemorrhage •CN palsy
3	Tumor biopsy	Tumor holding forceps, rongeur		Hemorrhage
4	Inferior pole resection and separation from lower cranial nerves and vessels	Microscope, facial nerve stimulator, bipolar, suction, microdissector, microscissors, Cottonoid patties, ultrasonic aspirator, tumor holding forceps	•Injury to CN IX, X, XI •Injury to vessels: AICA, PICA •Incomplete tumor excision	•CN IX, X, XI palsy •Hemorrhage •Infarct
5	Identification of CN VIII at brainstem and dissection of arachnoid medially	Microscope, facial nerve stimulator, bipolar, suction, microdissector, microscissors, Cottonoid patties, knife	•Incorrect arachnoid plane •Perforating vessel injury •Injury to CN VII or VIII	•Brainstem, peduncle infarct •CN VII palsy •Hearing loss if preservation intended
6	± Identification of dorsal cochlear nucleus for DNAP electrode if considering cochlear preservation	DNAP electrode		
7	Identification of the root entry of CN VII which lies ventral and inferior to root entry of CN VIII	Microscope, facial nerve stimulator, bipolar, suction, microdissector, microscissors, Cottonoid patties	•Vessel injury •Injury to CN VII	•Hemorrhage or infarct •CN VII palsy
8	± FREMAP electrode	FREMAP electrode		
9	Superior pole resection	Microscope, facial nerve stimulator, bipolar, suction, microdissector, microscissors, Cottonoid patties, ultrasonic aspirator, tumor holding forceps	•Injury to CN V or VII •Injury to petrosal vein or SCA •Incomplete tumor excision	•CN V, VII palsy •Hemorrhage •SCA infarct
10	Identification and protection of petrosal vein ± coagulation and division of petrosal vein only if absolutely necessary	Microscope, bipolar, suction, microdissector, microscissors, Cottonoid patties, scalpel	•Traction on petrosal vein •Injury to SCA •Sinus injury	•Venous infarct or hematoma •Air embolism •Sinus thrombosis
11	Dissection of tumor capsule from CN V	Microscope, facial nerve stimulator, bipolar, suction, microdissector, microscissors, Cottonoid patties	•Injury to CN IV or V •Injury to SCA	•CN IV or V palsy •SCA infarct
12	Medial to lateral dissection and rolling of the tumor from cerebellar peduncle and brain stem	Microscope, facial nerve stimulator, bipolar, suction, microdissector, microscissors, Cottonoid patties, tumor holding forceps, ultrasonic aspirator	•CN VII injury at root entry zone •Injury to perforating vessels •Incomplete tumor excision	•CN VII palsy •Peduncle or brainstem infarct
13	Drilling of internal auditory canal	Drill, irrigation, (± cutting, ± diamond burr), curette, bone wax, facial nerve stimulator	•Air cell opening without repair •Opening of the labyrinth or endolymphatic duct •Jugular bulb injury •Injury to CN VII or cochlear nerve	•Hearing loss •CSF leak •Hemorrhage •Air embolism •CN VII or cochlear nerve palsy
14	Incise dura of IAM and reflect away from tumor	Drill, irrigation, (± cutting, ± diamond burr), curette, bone wax, facial nerve stimulator	•CN injury •Vessel injury	•Hemorrhage •CN palsy
15	Locate fundus of IAM and dissect superior vestibular nerve as laterally as possible	Microscope, facial nerve stimulator, bipolar, suction, microdissector, microscissors, Cottonoid patties, knife	•Injury to CN VII or cochlear nerve •Incomplete tumor excision	•CN VII or cochlear nerve palsy
16	± Sacrifice of cochlear nerve in large tumors	Microscope, facial nerve stimulator, bipolar, suction, microdissector, microscissors, knife, blunt hook, facial nerve stimulator	•Failure to identify CN VII in distal canal as distinct from tumor and other CN •Injury to cochlear nerve in attempted hearing preservation surgery	•CN VII palsy •Hearing loss
17	Continue dissection with lateral to medial dissection to the porous	Microscope, facial nerve stimulator, bipolar, suction, microdissector, microscissors, Cottonoid patties, knife	•Failure to keep CN VII visualized at all times •Failure to maintain plane between tumor and CN VII •Incomplete tumor excision	•CN VII palsy
18	Resection of tumor in the CPA until lateral–medial and medial–lateral dissections to join together	Microscope, facial nerve stimulator, bipolar, suction, microdissector, microscissors, Cottonoid patties, ultrasonic aspirator, tumor holding forceps	•CN injury •Vessel injury •Incomplete tumor excision	•Hemorrhage •CN palsy
19	Removal of tumor after stepwise rolling and debulking of tumor as above	Microscope, facial nerve stimulator, bipolar, suction, microdissector, microscissors, Cottonoid patties, ultrasonic aspirator, tumor holding forceps	•CN injury •Vessel injury •Brainstem or peduncle injury •Incomplete tumor excision	•Hemorrhage •CN palsy •Brainstem or peduncle edema or infarct
20	± In circumstance when facial nerve is not preserved, perform facial nerve graft (proximal and distal stump anastomosis using nerve ± sural or greater auricular nerve)	Scalpel, monopolar, retractor, microscope, suture	•Incomplete anastomosis •CN injury	•CN VII palsy

Abbreviations: AICA, anterior inferior cerebellar artery; CN, cranial nerve; CPA, cerebellopontine angle; CSF, cerebrospinal fluid; DNAP, dorsal cochlear nucleus action potential; FREMAP, facial nerve root exit zone–elicited compound muscle action potential; IAM, internal auditory meatus; IQR, interquartile range; PICA, posterior inferior cerebellar artery; SCA, superior cerebellar artery.

Note: We appreciate the exact order of the following steps will be surgeon and tumor characteristic dependent.

#### Phase 3: Closure Phase

This phase consisted of nine steps, beginning with facial nerve stimulation, hemostasis, dural repair, and multilayer closure ( Table 4 ).

**Table 4:** Retrosigmoid operative workflow phase 3: closure

No.	Steps	Instruments	Technique error	Event error
1	CN VII stimulation to confirm response at low level (0.05 mA)	Facial nerve stimulator	•No stimulation	•CN VII palsy
2	Hemostasis	Bipolar, fibrin sealant, oxidized cellulose matrix, Cottonoid patties	•Incomplete hemostasis	•Hematoma
3	Seal mastoid air cells	Bone wax, fibrin glue	•Failure to seal mastoid air cells	•CSF leak
4	Resection cavity inspection		•Failure to identify residual tumor	•Recurrence or incomplete tumor resection
5	Dural repair	Suture, ± synthetic dural substitute, ± dural sealant glue, ± pericranium graft	•Incomplete closure	•CSF leak •Pseudomeningocoele
6	± Replacement of bone flap	Bone flap, miniplates, screws, ± bone substitute, ± bone flap clamp system	•Incomplete closure	•CSF leak •Pseudomeningocoele
7	± Replacement of bone dust or bone cement	Bone dust or bone cement	•Incomplete closure	•CSF leak
8	Closure of muscle layer and fascia	Suture	•Incomplete closure	•CSF leak •Infection
9	Skin closure	Suture, clips	•Poor opposition of skin edges	•CSF leak •Wound infection

Abbreviations: CFS, cerebrospinal fluid; CN, cranial nerve.

## Discussion

### Principal Findings

We present a consensus-derived codified operative workflow for retrosigmoid approach to vestibular schwannoma that considers the phases, steps, technique errors, and event errors of the operation. The operative workflow was achieved through national collaboration with the BSBS following an open invitation to all members to participate. This comprised 18 independently practicing neurosurgeons and ENT surgeons from 11 centers across the United Kingdom.

The retrosigmoid approach operative workflow comprised three distinct phases as follows: (1) approach and exposure, (2) tumor debulking and excision, and (3) closure, with a total of 40 individual steps. Participants felt strongly about protecting the petrosal vein and avoiding sacrifice if it all possible to prevent the risk of venous infarct or hemorrhage. Regarding tumor debulking and excision, the Phase 2, the aim is to achieve maximal tumor resection while preserving the facial nerve. As such, the tumor debulking and excision phase presented is an illustrative example. The exact sequence of resection is tumor and surgeon specific. We acknowledge that it is a systematic, stepwise debulking of the superior and inferior poles, and joining of medial–lateral and lateral–medial dissections but that depending on local experience and expertise, intraoperative findings, and the sequence of resection might differ from our operative workflow.

Vestibular schwannoma resection is a challenging surgery, with nuance relating to many aspects of the procedure. The retrosigmoid approach can be performed with the patient in a sitting, lateral, or supine position. Further, the incision and how to deal with the muscles during opening is important to avoid muscle atrophy or numbness. We acknowledge the heterogeneity in practice relating to local expertise and surgeon preference. The codified retrosigmoid approach presented in this manuscript can serve as foundational research for further work, such as operative workflow analysis or neurosurgical education.

#### Utilizing the Operative Workflow for Simulation and Education

Vestibular schwannomas cause unilateral hearing loss, tinnitus, imbalance, and headaches. 28 Zhang et al 29 reported a large retrospective series of 1,006 patients undergoing vestibular schwannoma resection. The mortality was 0.3%, risk of meningitis was 1.2%, and risk of cerebrospinal fluid (CSF) leak was in 9% of cases. Their reported CSF leak rate and the need for revision surgery decreased over time, while House–Brackmann facial nerve grade and hearing preservation after surgery improved over time. The authors cite improvement in functional outcomes that is due to increasing experience on smaller vestibular schwannomas. 29 In modern practice, smaller vestibular schwannomas are often managed nonsurgically with stereotactic radiosurgery. 4 This heralds an issue for current neurosurgical and ENT trainees, as there is a reduction in the number of smaller tumors to resect, train on, and enhance operative skills. Additionally, the learning curve for vestibular schwannoma resection is steep, 30 such that limited surgical experience in low-volume centers can result in poor functional outcomes and increased morbidity. 31 Adequate training and experience are essential in reducing mortality and morbidity. Operative workflows can provide a medium to explore and improve simulation, through the creation of high-fidelity models. 32 This can improve training experience and reduce mortality and morbidity.

Postoperative complications resulting from intraoperative errors during microsurgery for vestibular schwannoma resection are well known and have been well reported traditionally. 33 34 Seventy-five percent of errors within neurosurgery are deemed as preventable and technical in nature. 35 For example, injury to the venous sinuses or the cerebellar arteries can have devastating consequences for patients during vestibular schwannoma resection. 33 34 36 37 Our operative workflows contain information on technique errors and event errors, 38 paired with an exact sequence of phases and steps. This provides a framework for the development of high-fidelity models which encompass the importance of error awareness, avoidance, and management. This can be integrated with a model using augmented reality overlay to simulate a vessel injury and subsequent bleeding. 39 This gives the surgical trainee the opportunity to face and deal with intraoperative complications in a safe environment with no harm to patients. High-fidelity simulation models incorporating the operative workflows, as presented here, may become an integral component of surgical training in the future. For example, Realists spinal models ( *https://www.realists.de/realspine* ) already simulate bleeding and complications.

Models must be valid, and appropriate for the task. “Validity” comprises face validity (realism), content validity (usefulness as a training skill), and construct validity (experts perform better than novices). 40 To validate the content of a model simulating the retrosigmoid approach, there must be an agreed operative workflow with which to compare. The operative workflows presented in this study therefore offer a mean to validate the content of retrosigmoid surgical simulators. The codified operative workflow also provides the opportunity to generate a specific technical skills assessment for trainees, adapting traditional examples, such as the Objective Structured Assessment of Technical Skills (OSATS), which can be further used to examine construct validity of the model. The retrosigmoid approach in this codified operative workflow is related to vestibular schwannoma resection, but Phase-1 approach and exposure and Phase-3 closure, could be applicable to all retrosigmoid approaches to the cerebellopontine angle. Therefore, future research could explore the different pathologies to generate codified workflows for different skull base pathologies utilizing the retrosigmoid approach.

### Strengths and Limitations

This study presents the first consensus-derived operative workflow that considers and digitizes the phases, steps, technique errors, and event errors for the retrosigmoid to vestibular schwannoma resection. Our methodology follows the precedence of existing literature and includes national experts with many years of experience of performing such surgeries. The operative workflows provide a platform to further explore the complexity of vestibular schwannoma resection within an existing framework and common language. It also provides a framework in which to assess and validate “cadaver freed training models,” such as UpSurgeon's Retrosigmoid model ( *www.upsurgeon.com* ).

The operative workflows do not include some controversial aspects of vestibular schwannoma surgery, such as the indications for the approach, patient positioning, or intraoperative decision-making if aiming for subtotal resection. However, we took a constraint-based, pragmatic approach to create a foundational digitized operative workflow as the first stage in developing this operative workflow research. Although we reached consensus with colleagues from the BSBS, the operative workflows only reflect practice across the United Kingdom. Further collaboration with our European and international colleagues to generate worldwide consensus would broaden the scope of application.

## Conclusion

We present a national, multicenter, consensus-derived codified operative workflow for the retrosigmoid approach to vestibular schwannoma resection. The workflows provide a framework detailing the phases, steps, technical errors, and event errors. The codified retrosigmoid approach presented in this manuscript can serve as foundational research for future work, such as operative workflow analysis or neurosurgical simulation and education.
